# Arthralgia in children: the epidemiological study

**DOI:** 10.1186/1546-0096-9-S1-P144

**Published:** 2011-09-14

**Authors:** VA Malievskiy

**Affiliations:** 1 Bashkir State Medical University, Ufa, Russia

## Background

Arthralgia is a frequent complaint of children, and it may be a symptom of arthritis and non-inflammatory diseases.

## Aim

To determine the prevalence of joint pains in children of different ages.

## Methods

Four towns and two rural districts of the Republic of Bashkortostan (Russia) were chosen for the questionnaire. The total number of the childhood population was 50442 children under 17 years of age. Totally 43907 children or their parents were examined with the help of the screening questionnaire. The parents were questioned by medical nurses during consulting reception hours, or in kindergartens, or at school. The children aged between 11 and 17 years answered the question list on their own. The screening questionnaire included two questions:

1. Did you observe any pain in joints of your child?

2. Did you observe any swelling in joints of your child?

Four variants of the answer were offered:

A – never happened, B – happened, but does not disturb for the last year, C – happened before and disturbs for the last year, D – happened for the last year.

## Results

Complaints of arthralgia in the anamnesis or for the last year (answers B, C and D) were defined in 5490 children (12.5 %). The frequency of complaints of joint pains was higher in the girls (13.1 %), than in the boys (11.9 %, p < 0.001). More frequently (2562 children, 5.8 %) the joint pains were marked earlier, but for the last year they did not disturb (answer B). In 1923 children (14.4 %) the pains happened in the past and happen for the last year (answer C), in 1005 children (2.3 %) the pains happened for the last year (answer D). The frequency of complaints of joint pains was 5.8 % in the age between 4 and 6 years, and 13.3 % in the age between 7 and 11 years, and 16.6 % in the juvenile age (between 12 and 17 years of age).

754 children and/or their parents answered the question positively about the joint swelling (1.7 %). 368 children and/or their parents indicated that the joint swelling had appeared or remained for the last year (0.8 %).

## Conclusion

The frequency of complaints of joint pains was defined in 12.5 % of the children. The prevalence of arthralgia was higher in the boys, than in the girls, and it increases with the age. Most children with joint pains did not suffer from the inflammatory joint disease.

